# Genomic and Clinicopathological Characterization of a Reassortant HPAI H5N1 (Clade 2.3.4.4b) in an Endangered Cinereous Vulture (*Aegypius monachus*) in South Korea, 2026

**DOI:** 10.1155/tbed/2623621

**Published:** 2026-08-18

**Authors:** Chang-Gi Jeong, Seongwon Hwang, Taeyeong Jung, Su-Beom Chae, Tae-Nam Kim, Nchimunya Siamulonga, Serin Sim, Geonwoo Baek, Jun-Soo Park, Won-Il Kim, Jae-Ik Han, Sang-Ik Oh, Jae-Ku Oem

**Affiliations:** ^1^ College of Veterinary Medicine, Jeonbuk National University, Iksan 54596, Republic of Korea, cbnu.edu; ^2^ Biosafety Research Institute, Iksan 54596, Republic of Korea; ^3^ Jeonbuk Wildlife Center, Jeonbuk National University, Iksan 54596, Republic of Korea, cbnu.edu

## Abstract

Clade 2.3.4.4b highly pathogenic avian influenza viruses (HPAIVs) continue to circulate widely in East Asia and undergo frequent reassortment in wild birds. Raptors are regarded as spillover hosts that may be exposed through predation or scavenging, yet integrated clinicopathologic and genomic investigations in cinereous vultures remain limited. Here, we describe a fatal H5N1 HPAIV infection in a cinereous vulture (*Aegypius monachus*) found in South Korea on January 17, 2026. On presentation, the cinereous vulture showed severe neurologic dysfunction, including inability to stand, right‐sided head tilt with pathologic nystagmus, reduced oculocephalic and palpebral reflexes, and intermittent bilateral leg tremors. The cinereous vulture died within 2 days after rescue, and a complete necropsy was performed. Hematologic and biochemical testing revealed marked heterophil predominance, severe lymphopenia, mild monocytosis, and globulin values near the upper end of the reference interval. An oropharyngeal swab tested positive for influenza A virus, and a virus isolate, designated A/Cinereous_Vulture/Korea/26‐JBN47/2026(H5N1), was recovered in embryonated chicken eggs. Histopathology showed nonsuppurative encephalitis and necrotizing myocarditis, and influenza A nucleoprotein was detected immunohistochemically in neurons and cardiomyocytes. Tissue real‐time RT‐PCR showed the lowest cycle threshold value in the brain. Whole‐genome sequencing demonstrated that 26‐JBN47 belonged to clade 2.3.4.4b and contained a polybasic HA cleavage site (PLREKRRKR/GLF). Segment‐level phylogenetic analysis revealed a reassortant genome constellation comprising a maintained H5N1 backbone in HA, NA, and M; low PAIV (LPAIV)‐associated but H5N1‐incorporated PA and NP segments; flyway‐associated PB2 and NS segments; and a PB1 segment phylogenetically linked to regional LPAIV lineages. Molecular marker analysis identified multiple substitutions previously reported to be associated with receptor‐binding properties, polymerase‐related fitness, virulence, and host‐response modulation, whereas canonical PB2 mammalian‐adaptive markers were absent. These findings show that 26‐JBN47 was a reassortant clade 2.3.4.4b H5N1 HPAIV associated with systemic disease and clinicopathological findings consistent with neurotropic and cardiotropic infection in a cinereous vulture. They also support the potential value of scavenging raptors as sentinels of local or regional HPAIV circulation involving reassortant viruses in East Asia.

## 1. Introduction

Highly pathogenic avian influenza (HPAI) remains a major threat to animal health, poultry production, wildlife conservation, and public health because of its broad host range and capacity for transboundary spread [1]. H5 HPAI viruses (HPAIVs) of the A/goose/Guangdong/1/1996 (Gs/Gd) lineage have diversified extensively through continuous mutation and frequent genome reassortment, resulting in the emergence of multiple phylogenetic clades and neuraminidase (NA) subtype constellations [1–3]. Since 2014, clade 2.3.4.4 H5Nx viruses have driven recurrent epizootics in wild birds and poultry on a global scale [3, 4], and clade 2.3.4.4b has since become the dominant epidemic lineage, disseminating across Eurasia, Africa, North America, South America, and, more recently, Antarctica [5–8].

In South Korea, clade 2.3.4.4 H5 HPAIVs have been repeatedly detected in both wild birds and poultry since their first recognition in 2014, and clade 2.3.4.4b has dominated the most recent epidemic waves [4, 9, 10]. Following the introduction of clade 2.3.4.4b H5N8 viruses during the 2020–2021 winter season, additional reassortant H5 viruses continued to emerge, underscoring ongoing viral influx and local evolutionary diversification [10–12]. During the 2021–2022 season, clade 2.3.4.4b H5N1 viruses became widely established in wild birds and poultry, and gene constellation‐based genotyping by the Animal and Plant Quarantine Agency (APQA), Republic of Korea further showed that these viruses comprised multiple reassortant genotypes, designated 21G1–21G4 [4, 13]. In the subsequent 2022–2023 winter season, this diversification accelerated markedly: APQA‐based genomic classification identified 21 distinct H5N1 genotypes (22G0–22G20), whereas parallel phylogenetic studies using an alternative nomenclature resolved at least 16 genotypes (Kor22–23A–P), together indicating extensive reassortment‐driven genetic diversity in wild birds and poultry [14, 15]. This pattern persisted during the 2023–2024 and 2024–2025 seasons, when multiple clade 2.3.4.4b HPAIVs, including H5N1, H5N6, and H5N3, were detected in Korea [16–18].

The long‐distance dissemination and ongoing genetic changes in HPAIVs are closely associated with migratory birds, particularly wild waterfowl, which constitute the principal natural reservoirs of AIVs and provide ecological opportunities for both long‐range dispersal and reassortment among cocirculating viruses [4, 19, 20]. In East Asia, the East Asian–Australasian Flyway (EAAF) links breeding and staging areas in Mongolia and the Russian Far East to wintering and stopover sites in China, the Korean Peninsula, and Japan, thereby providing a major ecological framework for the repeated introduction, spread, and maintenance of HPAIVs in the region [21, 22]. In the Republic of Korea, the seasonal timing of HPAI detections and outbreaks typically coincides with the autumn‐to‐winter arrival of migratory waterfowl, further supporting the epidemiological importance of wild‐bird movements in viral incursion and onward transmission [9, 23].

Raptors represent a distinct exposure group within HPAIV ecology because infection in these species is thought to occur primarily through predation or scavenging on infected prey or carcasses rather than through maintenance in reservoir populations [24–26]. Telemetry‐based studies of the cinereous vulture (*Aegypius monachus*) have demonstrated seasonal movement of many juvenile and immature individuals between Mongolia and the Republic of Korea, where they overwinter, with broad and partially overlapping migration routes and home ranges [27, 28]. As an obligate scavenger, the cinereous vulture may be at increased risk of HPAIV exposure through scavenging on infected wild‐bird carcasses, particularly during seasonal movements across regions affected by avian influenza outbreaks [24, 29]. Moreover, because the cinereous vulture is globally classified as near threatened by the International Union for the Conservation of Nature (IUCN), HPAI infection in this species should be viewed not only as a matter of disease ecology but also as a conservation concern [30]. In South Korea, however, studies of HPAI in wild birds have focused predominantly on waterfowl and other commonly affected species.

In contrast, dedicated studies in raptors—and particularly in cinereous vultures—remain comparatively limited [9, 14, 31]. This gap is especially important because the clinical course, pathological lesions, and virological characteristics of HPAI infection in scavenging raptors may differ from those observed in traditional reservoir hosts or other wild‐bird taxa [24–26]. Accordingly, the present study aimed to characterize the clinicopathologic manifestations and viral genomic features of a fatal clade 2.3.4.4b H5N1 HPAIV infection in a cinereous vulture detected in South Korea. Rather than describing infection in a raptor host alone, this study combines species‐specific clinicopathologic findings with segment‐level genomic characterization to relate host disease manifestations to the reassortment constellation of the infecting virus.

## 2. Materials and Methods

### 2.1. Case History, Sampling, and Postmortem Examination

On January 17, 2026, a cinereous vulture (*A. monachus*) showing severe neurologic dysfunction was found in Sunchang‐gun, Jeonbuk State, Republic of Korea, and was rescued by the Jeonbuk National University Wildlife Rescue Center. Upon admission, the cinereous vulture underwent physical and neurologic examinations. Blood samples were collected for hematologic analysis, and an oropharyngeal swab was obtained for virological testing. The cinereous vulture died within 2 days after rescue, and a complete necropsy was subsequently performed. Samples from the brain, lung, liver, kidney, spleen, and small intestine were stored at −20°C for further analysis. Samples from the brain, heart, liver, and kidney were fixed in 10% neutral phosphate‐buffered formalin. The lung tissue sample was preserved in 99.9% ethanol.

### 2.2. Histopathological Evaluation

Formalin‐fixed tissues were washed under running tap water, and ethanol‐preserved lung tissue was rehydrated through a descending ethanol series prior to routine tissue processing. All tissues were dehydrated through ascending‐graded ethanol, cleared in xylene, and embedded in paraffin. Paraffin‐embedded tissues were sectioned at 3 μm, deparaffinized, rehydrated, and stained with hematoxylin and eosin (H&E) for histopathological evaluation.

Immunohistochemistry (IHC) was performed to detect the nucleoprotein of AIV. Antigen retrieval was conducted using citrate buffer (pH 6.0) at 98°C for 10 min, followed by cooling it at room temperature for 30 min. Sections were blocked with Super Block (ScyTek) for 20 min and then incubated overnight at 4°C with an anti‐influenza A virus nucleoprotein antibody (ab20343, Abcam, UK) at a dilution of 1:1000. Slides were subsequently incubated at room temperature for 1 h with a horseradish peroxidase‐conjugated secondary antibody (MP‐7500, Vector, USA). Immune complexes were visualized using the Vectastain DAB Substrate Kit (SK‐4105, Vector, USA) according to the manufacturer’s instructions, and the sections were counterstained with hematoxylin. All slides were examined with a light microscope (BX53, Olympus, Japan), and images were captured using a digital camera (DP80, Olympus, Japan).

### 2.3. Virological Detection, Virus Isolation, and Subtype Identification

Samples were screened for AIV using the LiliF AIV M real‐time reverse transcription polymerase chain reaction (rRT‐PCR) kit (iNtRON Biotechnology, Seongnam, Republic of Korea), as previously described [18]. For virus isolation, the AIV RNA‐positive oropharyngeal swab sample was diluted in 1× phosphate‐buffered saline (PBS; pH 7.4) supplemented with antibiotics (100 U/μL penicillin and streptomycin), centrifuged at 3000 ×*g* for 10 min, and filtered through a sterile 0.45‐μm membrane filter (GVS, Fairfield, NJ, USA). The clarified filtrate was then inoculated into 9‐ to 11‐day‐old specific‐pathogen‐free embryonated chicken eggs and incubated at 37°C for 3–4 days. Following incubation, allantoic fluid was harvested for viral RNA extraction using the Miracle‐AutoXT Automated Nucleic Acid Extraction System (iNtRON Biotechnology, Seongnam, Republic of Korea). AIV subtype identification was subsequently performed by RT‐qPCR using the TOPreal One‐step RT‐qPCR Kit (Enzynomics, Daejeon, Republic of Korea) according to previously described protocols [18, 32].

### 2.4. Whole‐Genome Sequencing and Molecular Marker Analysis

Viral RNA was extracted from AIV‐positive allantoic fluid and subjected to multisegment reverse transcription PCR, as previously described [33]. Sequencing was performed using the BIONICS index‐tagged (BIT) method (BIONICS, Republic of Korea), generating ~100,000 reads. Raw reads were quality‐filtered using Trimmomatic (v0.39), and genome assembly was performed with SPAdes (v3.15.4). The nucleotide sequences generated in this study were deposited in the EpiFlu database of the Global Initiative on Sharing All Influenza Data (GISAID) (Supporting Information, 1: Table S1).

Molecular markers associated with mammalian adaptation, pathogenicity, and antiviral drug resistance were identified using FluMut [34], an open‐source tool for mutation surveillance in highly pathogenic H5N1 genomes. Briefly, the nucleotide sequences generated in this study were analyzed with FluMut, which screens input H5N1 sequences against a curated marker database (FluMutDB) and reports mutations with previously described phenotypic relevance, including host adaptation, virulence‐associated changes, receptor‐binding alterations, replicative capacity, and antiviral resistance. The biological significance of the detected markers was interpreted based on FluMut output and its associated reference framework.

### 2.5. Sequence Dataset and Phylogenetic Analysis

Reference hemagglutinin (HA) gene sequences used for phylogenetic analysis were retrieved from the GISAID database. All available clade 2.3.4.4b H5 AIV HA sequences collected from 2020 onward were downloaded, together with the top 20 BLAST hits of the study isolate. To reduce dataset redundancy and improve computational efficiency, sequences were clustered by sequence identity using CD‐HIT (v4.8.1) [35]. The final dataset comprised 194 HA sequences, including the H5N1 virus identified in this study (Supporting Information 2: Table S2).

A maximum‐likelihood (ML) phylogenetic tree was constructed using FastTree v2.1.11 [36]. In addition, a time‐scaled maximum clade credibility (MCC) tree of the HA gene was reconstructed using BEAST v1.10.4 [37] under the Hasegawa–Kishino–Yano (HKY) substitution model, an uncorrelated log‐normal relaxed molecular clock, and a Gaussian Markov random field (GMRF) Bayesian skyride coalescent prior [38]. Four independent Markov chain Monte Carlo (MCMC) analyses were run for 100 million generations each, with sampling every 15,000 generations. The BEAGLE library was used to improve the computational performance. The convergence of the MCMC chains was assessed using Tracer v1.7.1 [39]. After burn‐in removal, the MCC tree was summarized using TreeAnnotator v1.10.4 and visualized in FigTree v1.4.2 (http://tree.bio.ed.ac.uk/software/figtree/).

Full‐genome sequences for each gene segment of AIVs isolated during 2020–2026 were retrieved from the GISAID database. Segment‐specific datasets were constructed using different inclusion criteria for subtypes. For the NA segment, HxN1 subtype sequences were included. For the remaining six internal gene segments (PB2, polymerase basic 2; PB1, polymerase basic 1; PA, polymerase acidic; NP, nucleoprotein; M, matrix; and NS, nonstructural), sequences representing all available subtype combinations were retrieved (e.g., H1N2, H3N8, and H9N2) (Supporting Information 2: Table S2). To reduce redundancy and minimize potential sampling bias, each segment dataset was clustered by sequence identity using CD‐HIT (v4.8.1). In addition, the top 20 sequences showing the highest similarity to the study isolate were included for each segment.

Multiple sequence alignments were generated for each segment using MAFFT software [40], with ~200–260 sequences included per analysis. Alignments were trimmed to the coding sequence region of each segment to exclude noncoding terminal regions and to enable comparisons across homologous coding regions. ML phylogenetic trees were reconstructed using RAxML‐NG (v1.2.0) [41] after selection of the best‐fit nucleotide substitution model with ModelFinder implemented in IQ‐TREE (v1.6.12) [42]. Branch support was evaluated using 1000 bootstrap replicates. Bootstrap values greater than 70% were displayed at the corresponding branch nodes and were used as supporting evidence for the relevant phylogenetic relationships. The resulting phylogenetic trees were visualized using the ggtree package [43] in R v4.3.3 [44]. Reassortment‐related relationships among the 26‐JBN47 gene segments were evaluated by comparing their segment‐specific phylogenetic placements.

## 3. Results

### 3.1. Case Presentation

At presentation, the cinereous vulture’s mentation was dull, and although there was intermittent resistance to external stimuli or restraint, it appeared slower and weaker compared with a clinically healthy state. The bird was unable to stand (incoordination of both wings and legs) and showed a right‐sided head tilt and intermittent tremors of both legs. Cranial nerve examination revealed the following extensive abnormalities: normal bilateral pupillary light reflexes but bilateral pathologic nystagmus, reduced palpebral and oculocephalic reflexes, and a reduced gag reflex. Conjunctival edema and congestion were also present, and regurgitated material in the oral cavity and diarrhea were noted.

Hematologic and plasma biochemical findings are summarized in Supporting Information 3: Table S3. When compared with published reference intervals for clinically healthy cinereous vultures [45], the total white blood cell count (16 × 10^3^ cells/μL) was within the reference interval, whereas marked heterophilia (15.36 × 10^3^ cells/μL; 96%) and lymphopenia (0.16 × 10^3^ cells/μL; 1%) were found. The monocyte count was mildly increased (0.48 × 10^3^ cells/μL; 3%). Erythrocyte indices were at the lower end of the reference interval, with a red blood cell count of 2.04 × 10^6^ cells/μL, a packed cell volume of 37%, and a hemoglobin concentration of 12.438 g/dL. Plasma biochemical analysis showed marked hyperglycemia (634 mg/dL), and globulin (3.4 g/dL) was near the upper end of the reference interval. The oropharyngeal swab was positive for AIV and was subsequently used for virus isolation in embryonated chicken eggs. The allantoic fluid harvested after inoculation showed hemagglutinating activity, with an HA titer of 512. Tissue real‐time RT‐PCR targeting the matrix (M) gene yielded the lowest Ct value in the brain (17.12), followed by the lung (27.67), small intestine (29.39), spleen (30.31), and liver (33.08).

### 3.2. Gross and Histopathological Lesions

**Figure 1:**
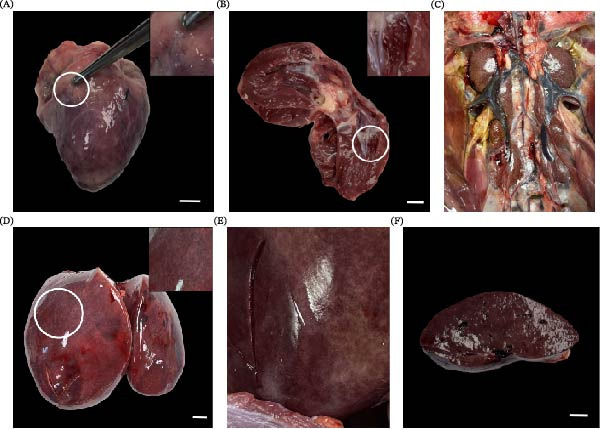
Representative gross pathological findings in the cinereous vulture naturally infected with clade 2.3.4.4b H5N1 HPAIV. (A) Heart. Mild petechial hemorrhages on the epicardial surface and (B) mild redness on the endocardial surface. (C) Mild renal congestion. (D, E) Enlarged, congested liver with multiple white foci on the surface. (F) Cross‐section of the liver showing congestion with compartmentalization. Insets show magnified views of the circled areas. Scale bars: 1 cm.

The necropsied cinereous vulture showed mild petechial hemorrhages on the epicardial surface (Figure 1A) and mild redness on the endocardial surface (Figure 1B). The kidneys showed mild congestion (Figure 1C). The liver was enlarged and congested, with multiple white foci on the surface (Figure 1D,E). A cross‐section of the liver also presented congestion lesions with compartmentalization (Figure 1F). No other gross lesions were observed in the brain, respiratory, digestive, and musculoskeletal systems.

**Figure 2:**
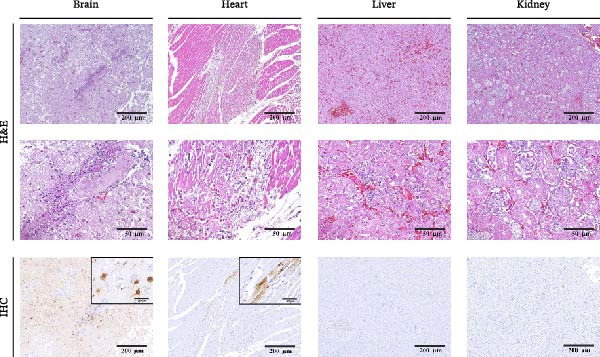
Histopathology and immunohistochemistry (IHC) for influenza A virus nucleoprotein in the major organ tissues from a cinereous vulture naturally infected with clade 2.3.4.4b H5N1 HPAIV. H&E‐stained sections from brain tissue showed multifocal neuronal necrosis and perivascular cuffing, and those from the heart presented lesions of cardiomyocyte necrosis with hemorrhage and inflammatory cell infiltration. In liver tissue, mild diffuse congestion, perivascular inflammatory cell infiltration, and hemosiderin deposition were observed; mild congestion was also observed in the kidney. Serial sections stained by IHC showed positive immunoreactivity for influenza A virus nucleoprotein in the brain and heart but were negative in liver and kidney tissue sections. Insets indicate higher magnification of IHC‐positive cells. Scale bars: 200 μm (top row and bottom row), 50 μm (middle row), and 20 μm (insets).

The representative histopathological lesions are shown in Figure 2. The cerebrum showed multifocal neuronal degeneration and necrosis with perivascular cuffing composed predominantly of mononuclear cells (lymphocytes) admixed with heterophils and macrophages. Neuronophagia, characterized by aggregates of microglial cells surrounding degenerating neurons, was present (Supporting Information 4: Figure S1A,B). Perivascular hemorrhage was also observed, along with perivascular inflammatory infiltration (Supporting Information, 4: Figure S1C). In the heart, focal cardiomyocyte necrosis with inflammatory cell infiltration was observed. No significant histopathological lesions were observed in the lung (Supporting Information 4: Figure S1D,E). The liver exhibited mild diffuse congestion, multifocal perivascular lymphocytic infiltration, and hemosiderin deposition. The kidney showed mild congestion, while tubular epithelial degeneration or necrosis lesions were not detected.

IHC for influenza A virus nucleoprotein revealed intranuclear and intracytoplasmic immunolabeling in numerous neurons and their processes, distributed multifocally within the cerebral cortex (Figure 2). In the heart, immunolabeling was detected in cardiomyocytes adjacent to lesions of myocardial necrosis and inflammatory cell infiltration. No immunoreactivity was observed in the lung (Supporting Information 4: Figure S1F), liver, or kidney.

### 3.3. Isolation and Whole‐Genome Sequencing of the Virus

The AIV RNA‐positive oropharyngeal sample yielded a virus isolate after inoculation into embryonated chicken eggs, as demonstrated by hemagglutinating activity in the harvested allantoic fluid. The isolate was designated A/Cinereous_Vulture/Korea/26‐JBN47/2026/H5N1 (hereinafter 26‐JBN47). Whole‐genome sequencing successfully recovered the complete coding sequences of all eight influenza A virus gene segments (PB2, PB1, PA, HA, NP, NA, M, and NS). The HA proteolytic cleavage site was identified as PLREKRRKR/GLF, which is consistent with the molecular hallmark of HPAIVs.

### 3.4. Genome Analysis

Time‐scaled phylogenetic analysis of the HA segment showed that the HA gene of 26‐JBN47 was closely related to clade 2.3.4.4b H5N1 viruses detected in East Asia during 2022–2023 and clustered within the G2c subgroup (Figure 3A). The two closest reference strains were A/duck/Miyagi/22B2T/2023(H5N1) and A/crow/Japan/TU‐19/2023(H5N1), both of which had a 100% nucleotide identity with 26‐JBN47 (Table 1). The time to the most recent common ancestor (tMRCA) of the HA cluster containing 26‐JBN47 and these two strains was estimated to be December 29, 2022 (95% highest posterior density [HPD]: November 10, 2022–January 26, 2023) (Figure 3B).

Figure 3Time‐scaled maximum clade credibility (MCC) tree of the HA gene showing the phylogenetic position of the clade 2.3.4.4b H5N1 isolate 26‐JBN47 recovered from a cinereous vulture. (A) Time‐scaled MCC tree reconstructed from HA gene sequences of clade 2.3.4.4b H5N1 viruses collected in Eurasia between 2020 and 2026. Major HA subgroups are indicated by shaded backgrounds and labeled as G2b, G2d, G2e, and G2c. Tip points are colored according to the country of detection. The horizontal axis represents the calendar year. (B) Expanded view of the G2c cluster highlighted by the red rectangle in (A). Black boxed labels indicate the estimated times to the most recent common ancestors (tMRCAs) of the relevant ancestral nodes, together with their corresponding 95% highest posterior density (HPD) intervals. Tip points are colored according to the country of detection. Scale bars indicate time in years.

**Figure:**
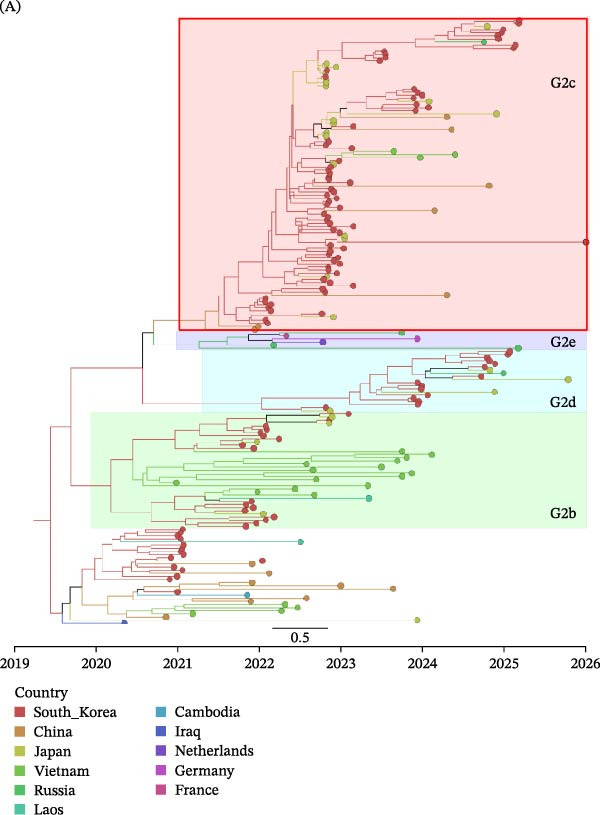

**Figure:**
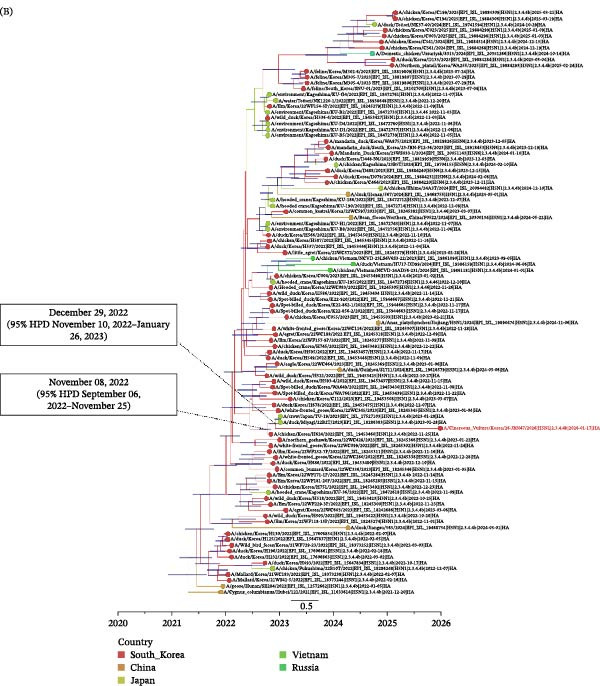

**Table 1:** Comparison of nucleotide sequence identities between segments of the 26‐JBN47 isolate and in the GISAID Epiflu database.

Segment	Virus name	Segment ID	Identity (%)
PB2	A/environment/Kagoshima/KU‐25‐H8/2025 (H5N1/2.3.4.4b HPAI)	EPI4899091	99.3
	A/environment/Kagoshima/KU‐25‐C5/2025 (H3N8/LPAI)	EPI4988807	98.7
PB1	A/environment/chongqing/1795/2023 (H9N2/LPAI)	EPI2841013	98.8
	A/mallard/Korea/KNU‐25/2023 (H1N1/LPAI)	EPI2873292	98.7
PA	A/environment/Kagoshima/KU‐25‐H8/2025 (H5N1/2.3.4.4b HPAI)	EPI4899090	99.5
	A/mallard/Korea/KNU‐25/2023 (H1N1/LPAI)	EPI2873289	98.1
HA	A/duck/Miyagi/22B2T/2023 (H5N1/2.3.4.4b HPAI)	EPI2752262	100.0
	A/crow/Japan/TU‐19/2023 (H5N1/2.3.4.4b HPAI)	EPI2528186	100.0
NP	A/duck/Korea/D051/2025 (H5N1/2.3.4.4b HPAI)	EPI4393694	99.1
	A/Wild_bird/South_Korea/E23‐931/2023 (H6N8/LPAI)	EPI3040224	99.0
NA	A/environment/Kagoshima/KU‐25‐I1/2025 (H5N1/2.3.4.4b HPAI)	EPI4787281	99.2
	A/environment/Kagoshima/KU‐25‐H8/2025 (H5N1/2.3.4.4b HPAI)	EPI4899093	99.1
M	A/turkey/Tyumen/81‐96V/2021 (H5N1/2.3.4.4b HPAI)	EPI4598814	99.1
	A/Falco_peregrinus/Beijing/1/2022 (H5N1/2.3.4.4b HPAI)	EPI2506575	99.1
NS	A/environment/Kagoshima/KU‐25‐H8/2025 (H5N1/2.3.4.4b HPAI)	EPI4899088	99.5
	A/wild_duck/Korea/H521/2022 (H5N1/2.3.4.4b HPAI)	EPI3581875	99.2

Phylogenetic analysis of the NA segment showed that 26‐JBN47 clustered most closely with A/environment/Kagoshima/KU‐25‐I1/2025(H5N1), A/environment/Kagoshima/KU‐25‐H8/2025(H5N1), and A/Env/Changsha/31/2023(H5N1) (Supporting Information 5: Figure S2 and Table 1).

Phylogenetic analysis of the PB2 segment showed that 26‐JBN47 clustered with A/environment/Kagoshima/KU‐25‐H8/2025(H5N1) and was closely associated with A/environment/Kagoshima/KU‐25‐C5/2025(H3N8) and A/environment/Kagoshima/KU‐25‐5b/2025(H3N8) in the corresponding tree. These related viruses were all collected in Kagoshima, Japan, during November 17–24, 2025 (Supporting Information 5: Figure S2 and Table 1). Thus, the PB2 segment was phylogenetically closely related to East Asian AIVs detected in both H5N1 and non‐H5 subtype backgrounds.

Phylogenetic analysis of the PB1 segment showed that 26‐JBN47 was most closely related to A/environment/chongqing/1795/2023(H9N2), A/mallard/Korea/KNU‐25/2023(H1N1), and A/bean_goose/Jiangsu/3‐1‐207/2022(H11N1). These top‐ranking related strains were all low PAIVs (LPAIVs) collected between December 2022 and March 2023 (Supporting Information 5: Figure S2 and Table 1).

For the PA segment, the closest related strain was A/environment/Kagoshima/KU‐25‐H8/2025(H5N1). Nearby branches also included LPAIVs, including A/Anser_fabalis/Jiangsu/G867/2023(H4N6) and A/mallard/Korea/KNU‐25/2023(H1N1), indicating that the PA segment clustered within the H5N1 lineage but remained closely connected to viruses of other subtypes (Supporting Information 5: Figure S2 and Table 1).

The NP segment was most closely related to A/duck/Korea/D051/2025(H5N1), with a 99.1% nucleotide identity. In the same phylogenetic tree, 26‐JBN47 was also placed near A/Wild_bird/South_Korea/E23‐931‐1/2023(H6N8) and A/Wild_bird/South_Korea/E23‐931/2023(H6N8), which formed adjacent branches within the broader cluster (Supporting Information 5: Figure S2 and Table 1). Thus, the NP segment was most closely related to a Korean H5N1 virus detected in 2025 but was also positioned near East Asian LPAIVs.

For the M segment, 26‐JBN47 also clustered with 2.3.4.4b HPAI H5N1 viruses detected in Eurasia during 2022–2023, including A/turkey/Tyumen/81‐96V/2021(H5N1), A/Falco_peregrinus/Beijing/1/2022(H5N1), and A/Spot‐billed_duck/Korea/K22‐862‐1/2022(H5N1). These viruses originated from Russia, China, and the Republic of Korea (Supporting Information 5: Figure S2 and Table 1). Together with the HA and NA results, the M segment clustered with previously detected East Asian or Eurasian H5N1 viruses.

In the NS phylogeny, 26‐JBN47 showed the highest nucleotide identity (99.5%) to A/environment/Kagoshima/KU‐25‐H8/2025(H5N1) and clustered with South Korean H5N1 viruses detected in 2022, including A/wild_duck/Korea/H521/2022(H5N1) and A/duck/Korea/H515/2022(H5N1). Nearby branches in the NS phylogeny also included non‐H5N1 subtype viruses (Supporting Information 5: Figure S2 and Table 1). Together, these results indicate that the NS segment was phylogenetically related to East Asian/Eurasian AIVs detected across multiple years and subtype backgrounds.

### 3.5. Molecular Marker Analysis

Molecular marker screening identified several amino acid substitutions that have been previously reported to be associated with receptor‐binding properties, polymerase activity, virulence, or host‐response modulation in experimental or surveillance‐based studies. In HA, S133A, T156A, K218Q/S223R, and S107R/T108I were identified, which are associated with receptor‐binding properties or virulence. Multiple substitutions previously linked to polymerase‐related phenotypes were also present. In PB2, K389R and V598T were identified, together with the broader constellation L89V/G309D/T339K/R477G/I495V/K627E/A676T. In PB1, D3V and D622G were present, whereas PA contained S37A, N383D, and N409S. Additional substitutions previously reported in relation to virulence or host‐response modulation were identified in the M and NS segments, including N30D, I43M, and T215A in M and P42S, I106M, C138F, the ESEV motif, L103F/I106M, and C138F/K55E/K66E in NS. Importantly, the canonical PB2 mammalian‐adaptive substitutions Q591K, E627K, and D701N were not detected in 26‐JBN47. The detected molecular markers and their reported associations are summarized in Table 2.

**Table 2:** Previously reported amino acid substitutions of potential functional relevance identified in 26‐JBN47.

Segment	Mutation	26‐JBN47	Associated effect	Reference
PB2	K389R	R	Increased polymerase activity and replication in mammalian cells	[46, 47]
	Q591K	Q	Increased replication in mammalian cells	[48]
	V598T	T	Increased polymerase activity and replication in mammalian cells Increased virulence in mice	[46, 47]
	E627K	E	Mammalian host adaptation, increased replication at lower temperature (33°C), and increased virulence	[49]
	D701N	D	Enhanced nuclear import via importin‐α binding, increased replication in mammals	[50]
	L89V, G309D, T339K, R477G, I495V, K627E, A676T	89V, 309D, 339K, 477G, 495V, 627E, 676T	Increased polymerase activity in mammalian cells Increased virulence in mice	[47, 51]

PB1	D3V	V	Increased polymerase activity in mammalian cells Increased replication in avian cells	[47, 52]
	D622G	G	Increased polymerase activity and virulence in mice	[47]

PA	S37A	A	Increased polymerase activity in mammalian cells	[47, 53]
	N383D	D	Increased polymerase activity in avian and mammalian cells	[47, 54, 55]
	N409S	S	Increased polymerase activity and replication in mammalian cells	[47, 56]

HA (H5 numbering)	S133A	A	Increased pseudovirus binding to α2–6	[47]
	T156A	A	Increased transmission in guinea pigs Increased virus binding to α2–6	[47, 57, 58]
	Q222L	Q	Shift from avian (α2–3) to human (α2–6) receptor binding	[59]
	S107R, T108I	107R, 108I	Increased pH of fusion Increased virulence in chickens Increased virulence in mice	[47, 60]
	K218Q, S223R	218Q, 223R	Increased virus binding to α2–3 and α2–6	[47, 61]

M	N30D	D	Increased virulence in mice	[47, 53]
	I43M	M	Increased virulence in mice	[47, 62]
	T215A	A	Increased virulence in mice	[47, 53]

NS	P42S	S	Decreased antiviral response in mice Increased virulence in mice	[47, 63]
	I106M	M	Increased viral replication in mammalian cells Increased virulence in mice	[47, 64]
	C138F	F	Decreased interferon response Increased viral replication in mammalian cells	[47, 65]
	80–84 DEL	TIASV^a^	Enhanced virulence in chickens and mice	[66]
	ESEV	ESEV	Increased virulence in mice	[67]
	L103F, I106M	103F, 106M	Increased virulence in mice	[47, 68]
	C138F, K55E, K66E	138F, 55E, 66E	Decreased interferon response Enhanced replication in mammalian cells	[47, 65]

^a^TIASV indicates that the wild‐type sequence is retained and the 5‐amino acid deletion is absent in 26‐JBN47.

## 4. Discussion

The hematologic and plasma biochemical findings were notable when compared with species‐specific reference intervals for clinically healthy cinereous vultures [45]. The most relevant abnormalities were marked heterophilia, lymphopenia, and globulin values near the upper end of the reference interval. Although the total white blood cell count remained within the reference interval, this observation remains compatible with an acute or rapidly progressive inflammatory response rather than with clinical normality. In addition, the globulin concentration, a positive acute‐phase protein, was near the upper limit of the reference interval. It is considered together with marked heterophilia, which supports the presence of an acute inflammatory response. These clinicopathologic changes occurred together with clear neurologic dysfunction at presentation, detection of HPAIV from the oropharyngeal swab, gross and microscopic lesions, and the highest viral RNA load in the brain. Taken together, these findings support acute H5N1‐associated systemic disease in this bird and indicate that the infection was clinically associated with neurologic dysfunction. These overall findings are consistent with previous reports of clade 2.3.4.4b H5N1 infection in Korean wild birds and naturally infected raptors [31, 69–71].

Histopathologically, the brain showed nonsuppurative encephalitis with perivascular cuffing and neuronophagia, and influenza A nucleoprotein was detected immunohistochemically in neurons and their processes. In addition, the lowest Ct value among all tissues examined indicates a high viral RNA burden in the brain, consistent with prominent central nervous system involvement following systemic infection. Concurrently, necrotizing myocarditis with immunohistochemical detection of viral antigens in cardiomyocytes indicates direct cardiac involvement. Together, these findings are consistent with neurotropic and cardiotropic infections in this cinereous vulture. Similar brain and heart involvement has also been reported in previous studies of clade 2.3.4.4b H5N1 infection in raptors, including bald eagles, red‐tailed hawks, great horned owls, and white‐tailed eagles [31, 69, 70]. In these species, encephalitis and myocarditis were predominant lesions, and viral antigen was mainly localized to the brain and heart. Similarly, experimental studies in white‐tailed sea eagles have shown that viral replication intensity in the brain correlates with disease severity and mortality, further supporting the relevance of brain involvement during clade 2.3.4.4b H5N1 infection in raptors [71]. Overall, the distribution of lesions and viral antigen in the present case is consistent with neurotropic and cardiotropic findings described in raptors infected with clade 2.3.4.4b HPAIV.

The HA, NA, and M segments formed the most stable H5N1‐associated component of the genome in 26‐JBN47. In the segment‐specific phylogenies, these three segments clustered with the epidemic East Asian H5N1 viruses detected from 2022 to 2023. Consistent with these results, recent studies from South Korea and Japan have shown that clade 2.3.4.4b viruses retain a recognizable H5‐associated backbone despite the substantial reassortment of internal gene segments [72, 73]. A similar genome constellation was also reported in the South Korean feline H5N1 study, in which the G10‐HPAIV origin gene segments (HA, NA, and M) were conserved, whereas the Eurasian LPAIV–origin internal segments differed among genotypes [74]. In the current study, HA/NA/M segments were clustered with G10‐like viruses, indicating that the clade 2.3.4.4b H5N1 backbone has been continuously maintained within wild‐bird populations in East Asia since 2022–2023.

**Figure 4:**
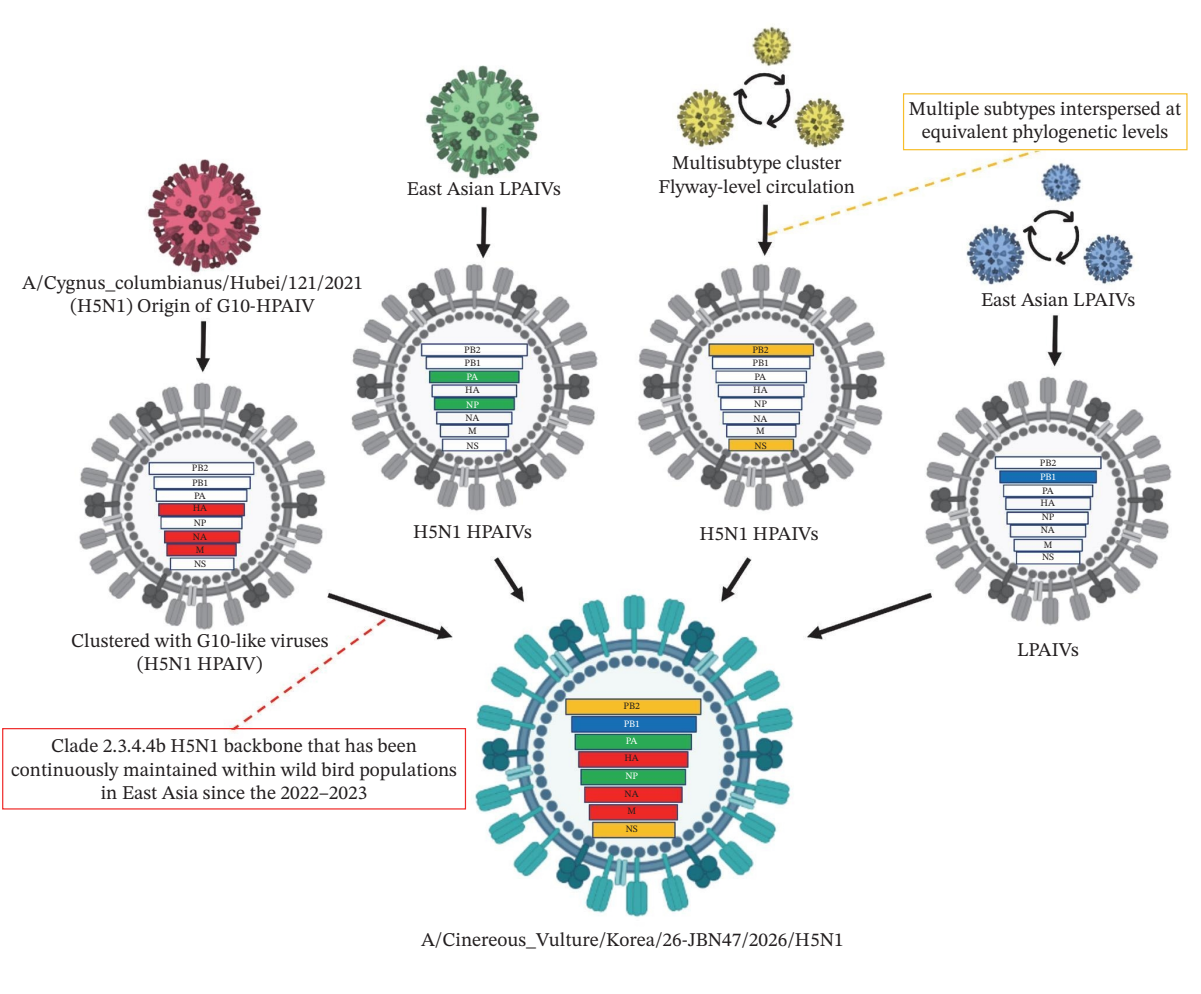
Schematic representation of the inferred reassortment‐related genome constellation of the cinereous vulture‐derived H5N1 isolate 26‐JBN47 from South Korea, January 2026. The schematic summarizes the segment‐specific phylogenetic relationships of 26‐JBN47 with H5N1 HPAIV‐ and East Asian LPAIV‐related gene segment pools based on the currently available sequence dataset. Bars represent the eight gene segments of avian influenza A virus in the following order from top to bottom: PB2, PB1, PA, HA, NP, NA, M, and NS. Different bar colors indicate segment groups with distinct phylogenetic relationships estimated from the segment‐specific phylogenetic trees. This diagram provides a phylogeny‐based overview of the genome constellation of 26‐JBN47.

The PA and NP segments remained within predominantly H5N1‐associated clusters despite visible links to regional LPAIV‐related branches, suggesting that these segments may already have been incorporated into circulating H5N1 genomes before the emergence of 26‐JBN47. By contrast, the PB2 and NS segments showed a broader flyway‐level circulation signal. For PB2, closely related H5N1 and H3N8 viruses were identified in Kagoshima over the same short sampling period. This finding suggests the continued circulation of the related PB2 segment through multiple subtypes in wild birds. The NS segment showed a similar pattern, clustering most closely with recent H5N1 viruses, while nearby branches still included other LPAIVs detected in different East Asian countries. Consistent with previous studies [17, 72, 73, 75], PB2 and NS appear to have circulated along the East Asian migratory flyway, with repeated incorporation into different genome constellations across multiple seasons. In contrast, the PB1 segment did not cluster within a contemporary H5N1‐dominated group. Rather, it showed its closest phylogenetic relationships to regional non‐H5 subtype LPAIVs, suggesting LPAIV‐related ancestry or prior reassortment involving regional AIV gene pools. However, because segment‐specific tMRCA analyses were not performed for PB1 or the other internal genes, the timing of this event cannot be determined from the present data alone. Taken together, 26‐JBN47 showed a four‐part genome constellation consisting of backbone‐maintained H5N1 segments (HA, NA, and M), LPAIV‐connected but H5N1‐incorporated segments (PA and NP), flyway‐associated circulating segments (PB2 and NS), and a PB1 segment phylogenetically linked to regional LPAIV lineages (Figure 4). This constellation reflects the segment‐specific phylogenetic relationships observed in the currently available sequence dataset and should be interpreted as an inferred genomic configuration rather than a definitive reconstruction of the reassortment pathway.

The molecular markers of 26‐JBN47 remained broadly consistent with contemporary avian‐origin clade 2.3.4.4b H5N1 viruses circulating in East Asia [74, 76], while also including several substitutions of potential biological relevance. The isolate retained an HPAI‐type HA cleavage motif, and multiple substitutions previously associated with receptor‐binding properties, polymerase activity, virulence, or host‐response modulation were identified (Table 2). However, the phenotypic effects of these substitutions may vary according to the subtype, viral backbone, host species, and gene constellation. Therefore, they should be interpreted as putative markers of potential functional relevance rather than definitive indicators of mammalian adaptation. Importantly, 26‐JBN47 lacked the canonical PB2 mammalian‐adaptive substitutions E627K, D701N, and Q591K. Together, these findings indicate that 26‐JBN47 carried several previously reported molecular markers of potential functional interest, but its marker profile did not support advanced mammalian adaptation.

Cinereous vultures are wide‐ranging scavenging raptors, and many immature birds overwinter in South Korea and move seasonally between the Korean Peninsula and Mongolia, with broad home ranges and overlapping movement routes across East Asia [27, 28]. Under these conditions, cinereous vultures would have more opportunities to be exposed to HPAIV, particularly through scavenging and repeated use of wintering habitats during periods of intense HPAIV circulation in wild birds [25, 26]. This pattern is consistent with recent evidence that predatory and scavenging birds are frequently affected during clade 2.3.4.4b H5N1 epizootics, as well as the recent H5N1 case reported in a wild leopard cat in South Korea [25, 76]. Accordingly, the present case is more consistent with infection in an ecologically exposed spillover host than with infection in a maintenance host. The absence of reports of an identical genome constellation in other hosts further supports this interpretation, although it does not exclude the possibility that related viruses circulated undetected in wild‐bird populations. Scavenging raptors are highly susceptible to HPAIV infection and are often detected only after overt disease or death rather than during subclinical carriage [25, 27, 28]. Given that the gene segments of 26‐JBN47 were phylogenetically linked to East Asian or Eurasian AIV gene pools, the reassortant constellation may have arisen either in local wintering habitats in South Korea or within migratory bird populations along the East Asian flyway before detection in this vulture. However, the precise location and timing of reassortment cannot be determined from the currently available sequence data. In addition, subclinical infections in wild waterfowl may be underrecognized because surveillance intensity varies by host species, geographic region, and sampling period. For this reason, findings in such hosts may provide an early indication of local HPAIV circulation around carrion sources, wintering habitats, or mixed wild‐bird interfaces. Thus, cinereous vultures may serve as potential sentinels of local or regional HPAIV circulation and the presence of reassortant viruses in East Asian wildlife systems.

## 5. Conclusion

This study documents a fatal clade 2.3.4.4b H5N1 HPAIV infection in a cinereous vulture in South Korea and links severe clinicopathologic disease with a reassortant viral genome. The affected cinereous vulture developed marked neurologic dysfunction and showed nonsuppurative encephalitis, necrotizing myocarditis, and viral antigen distribution in the brain and heart, with the highest viral RNA burden detected in the brain. Together, these findings are consistent with neurotropic and cardiotropic infections in this case. Genomic analyses showed that 26‐JBN47 retained an East Asian H5N1 backbone in the HA, NA, and M segments, which clustered with G10‐like viruses, whereas the internal genes reflected multiple reassortment histories involving regional AIV gene pools. The molecular marker profile was broadly consistent with contemporary avian‐origin clade 2.3.4.4b H5N1 viruses and did not indicate advanced mammalian adaptation. These findings show that cinereous vultures can develop severe fatal diseases after HPAIV infection and support their potential utility as sentinels of local or regional HPAIV circulation involving reassortant viruses near wildlife interfaces in East Asia.

## Author Contributions

**Chang-Gi Jeong**: conceptualization, data curation, formal analysis, investigation, visualization, validation, writing – original draft. **Seongwon Hwang**: investigation, methodology, formal analysis, visualization. **Taeyeong Jung**: investigation, methodology. **Su-Beom Chae and Tae-Nam Kim**: investigation, data curation. **Nchimunya Siamulonga**, **Serin Sim**, **Geonwoo Baek** **, and Jun-Soo Park**: investigation. **Won-Il Kim and Sang-Ik Oh**: validation. **Jae-Ik Han**: resources, validation. **Jae-Ku Oem**: project administration, conceptualization, supervision, writing – review and editing.

## Funding

This research was supported by the Bio & Medical Technology Development Program of the National Research Foundation (NRF), funded by the Ministry of Science and ICT (MSIT), Republic of Korea (Grant RS‐2023‐00228644), and by the Ministry of Education (Grant 2019R1A6A1A03033084).

## Ethics Statement

Ethical review and approval were not required for this study because no animals were captured, purchased, or experimentally infected for research purposes. The cinereous vulture described in this study was a free‐ranging wild bird rescued and admitted for clinical care. Diagnostic samples were collected as part of routine veterinary practice, and subsequent analyses were conducted using the diagnostic and preserved samples. Necropsy and tissue sampling were performed after spontaneous death for diagnostic purposes. All procedures, including sample use and carcass handling, complied with national regulations and biosafety guidelines, and no specific permits were required.

## Conflicts of Interest

The authors declare no conflicts of interest.

## Supporting Information

Additional supporting information can be found online in the Supporting Information section.

## Supporting information


**Supporting Information 1** Table S1: Sequence information for the cinereous vulture‐derived H5N1 virus isolated in this study.

**Supporting Information 2** Table S2: List of influenza A viruses used for maximum‐likelihood tree construction.

**Supporting Information 3** Table S3: Hematological and plasma biochemical findings of the cinereous vulture infected with H5N1.

**Supporting Information 4** Figure S1: Representative histopathological and immunohistochemical findings in the brain and lung. (A, B) Brain, H&E staining. Neuronophagia, characterized by aggregates of microglial cells (small, dark nuclei) surrounding a degenerating neuron (arrows). (C) Brain, H&E staining. Perivascular hemorrhage with inflammatory cell infiltration at the margins of the hemorrhagic focus. (D, E) Lung, H&E staining. The pulmonary parenchyma showed no significant inflammatory or necrotizing lesions. The observed changes were limited to postmortem hypostatic blood accumulation within vessels (D) and preservation‐related artifacts, attributable to prolonged storage in 99.9% ethanol (E). (F) Lung, immunohistochemistry for influenza A virus nucleoprotein. No specific immunolabeling was detected. Scale bars: 50 (A, B, E), 100 (C), and 200 μm (D, F).

**Supporting Information 5** Figure S2: Maximum‐likelihood (ML) phylogenetic trees of the seven non‐HA gene segments of the cinereous vulture‐derived H5N1 isolate 26‐JBN47. ML trees were reconstructed for the NA, M, PA, NP, PB2, NS, and PB1 segments of 26‐JBN47 using Eurasian avian influenza virus sequences. The 26‐JBN47 isolate is highlighted in a blue rectangle and text. Colored circles at the tips of the tree denote the country of detection. Annotation tiles indicate, from left to right, genotype, lineage, clade, and nucleotide identity (%) relative to the corresponding segment of 26‐JBN47. The scale bar indicates nucleotide substitutions per site.

## Data Availability

The nucleotide sequences generated in this study were deposited in the GISAID EpiFlu database and are listed in Supporting Information 1: Table S1. The influenza A virus sequences used for phylogenetic analyses are listed in Supporting Information 2: Table S2.

## References

[1] King J. , Harder T. , Conraths F. J. , Beer M. , and Pohlmann A. , The Genetics of Highly Pathogenic Avian Influenza Viruses of Subtype H5 in Germany, 2006–2020, Transboundary and Emerging Diseases. (2021) 68, no. 3, 1136–1150, 10.1111/tbed.13843. 32964686

[2] Lee D.-H. , Bertran K. , Kwon J.-H. , and Swayne D. E. , Evolution, Global Spread, and Pathogenicity of Highly Pathogenic Avian Influenza H5Nx Clade 2.3.4.4, Journal of Veterinary Science. (2017) 18, no. S1, 269–280, 10.4142/jvs.2017.18.S1.269. 28859267 PMC5583414

[3] Claes F. , Morzaria S. P. , and Donis R. O. , Emergence and Dissemination of Clade 2.3.4.4 H5Nx Influenza Viruses—How Is the Asian HPAI H5 Lineage Maintained, Current Opinion in Virology. (2016) 16, 158–163, 10.1016/j.coviro.2016.02.005. 26991931

[4] Kim J.-Y. , Jeong S. , and Kim D.-W. , et al.Genomic Epidemiology of Highly Pathogenic Avian Influenza A (H5N1) Virus in Wild Birds in South Korea During 2021-2022: Changes in Viral Epidemic Patterns, Virus Evolution. (2024) 10, no. 1, 10.1093/ve/veae014. PMC10919474 38455682

[5] Fusaro A. , Zecchin B. , and Giussani E. , et al.High Pathogenic Avian Influenza a(H5) Viruses of Clade 2.3.4.4b in Europe—Why Trends of Virus Evolution are More Difficult to Predict, Virus Evolution. (2024) 10, no. 1, 10.1093/ve/veae027. PMC11065109 38699215

[6] Banyard A. C. , Bennison A. , and Byrne A. M. P. , et al.Detection and Spread of High Pathogenicity Avian Influenza Virus H5N1 in the Antarctic Region, Nature Communications. (2024) 15, no. 1, 10.1038/s41467-024-51490-8, 7433. PMC11372179 39227574

[7] Lewis N. S. , Banyard A. C. , and Whittard E. , et al.Emergence and Spread of Novel H5N8, H5N5 and H5N1 Clade 2.3.4.4 Highly Pathogenic Avian Influenza in 2020, Emerging Microbes & Infections. (2021) 10, no. 1, 148–151, 10.1080/22221751.2021.1872355. 33400615 PMC7832535

[8] Lee D.-H. , Torchetti M. K. , and Winker K. , et al.Intercontinental Spread of Asian-Origin H5N8 to North America Through Beringia by Migratory Birds, Journal of Virology. (2015) 89, no. 12, 6521–6524, 10.1128/JVI.00728-15. 25855748 PMC4474297

[9] Lee S.-H. , Kwon J.-H. , Youk S. , Lee S.-W. , Lee D.-H. , and Song C.-S. , Epidemiology and Pathobiology of H5Nx Highly Pathogenic Avian Influenza in South Korea (2003–2024): A Comprehensive Review, Veterinary Quarterly. (2025) 45, no. 1, 23–38, 10.1080/01652176.2025.2498918. 40332021 PMC12064103

[10] Baek Y. G. , Lee Y. N. , and Lee D. H. , et al.Multiple Reassortants of H5N8 Clade 2.3.4.4b Highly Pathogenic Avian Influenza Viruses Detected in South Korea During the Winter of 2020–2021, Viruses. (2021) 13, no. 3, 10.3390/v13030490, 490. 33809549 PMC8001867

[11] Kang Y.-M. , Heo G.-B. , and An S.-H. , et al.Introduction of Multiple Novel High Pathogenicity Avian Influenza (H5N1) Virus of Clade 2.3.4.4b Into South Korea in 2022, Transboundary and Emerging Diseases. (2023) 2023, 10.1155/2023/8339427, 8339427. 40303677 PMC12017251

[12] Sagong M. , Lee Y.-N. , and Song S. , et al.Emergence of Clade 2.3.4.4b Novel Reassortant H5N1 High Pathogenicity Avian Influenza Virus in South Korea During Late 2021, Transboundary and Emerging Diseases. (2022) 69, no. 5, e3255–e3260, 10.1111/tbed.14551. 35413157

[13] Cha R. M. , Lee Y.-N. , and Park M.-J. , et al.Genetic Characterization and Pathogenesis of H5N1 High Pathogenicity Avian Influenza Virus Isolated in South Korea During 2021–2022, Viruses. (2023) 15, no. 6, 10.3390/v15061403, 1403. 37376703 PMC10304347

[14] Seo Y.-R. , Cho A. Y. , and Si Y.-J. , et al.Evolution and Spread of Highly Pathogenic Avian Influenza A(H5N1) Clade 2.3.4.4b Virus in Wild Birds, South Korea, 2022–2023, Emerging Infectious Diseases. (2024) 30, no. 2, 299–309, 10.3201/eid3002.231274. 38215495 PMC10826760

[15] Cha R. M. , Park M.-J. , and Baek Y.-G. , et al.Genetic Characteristics and Pathogenesis of Clade 2.3.4.4b H5N1 High Pathogenicity Avian Influenza Virus Isolated From Poultry in South Korea, 2022–2023, Virus Research. (2025) 353, 10.1016/j.virusres.2025.199541, 199541. 39894372 PMC11850744

[16] Heo G.-B. , Kang Y.-M. , and An S.-H. , et al.Concurrent Infection With Clade 2.3.4.4b Highly Pathogenic Avian Influenza H5N6 and H5N1 Viruses, South Korea, 2023, Emerging Infectious Diseases. (2024) 30, no. 6, 1223–1227, 10.3201/eid3006.240194. 38703023 PMC11138991

[17] Cha R. M. , Jang Y. , and Park M.-J. , et al.Emergence and Genetic Characteristics of H5N1, H5N6, and H5N3 Clade 2.3.4.4b High Pathogenicity Avian Influenza Viruses in South Korea During the 2023–2024 and 2024–2025 Winter Seasons, Transboundary and Emerging Diseases. (2026) 2026, no. 1, 10.1155/tbed/8053623, 8053623. 41743081 PMC12931157

[18] Jeong C. G. , Lee C. Y. , and Chae S. B. , et al.Emergence of HPAI H5N6 Clade 2.3.4.4b in Wild Birds: A Case Study From South Korea, Transboundary and Emerging Diseases. (2024) 2023, 4141478. 10.1155/tbed/4141478 PMC12020246 40303050

[19] Couty M. , Guinat C. , and Fornasiero D. , et al.The Role of Wild Birds in the Global Highly Pathogenic Avian Influenza H5 Panzootic, 2020–2023, npj Biodiversity. (2026) 5, no. 1, 10.1038/s44185-025-00114-5, 1. 41501345 PMC12780116

[20] Yang Q. , Wang B. , and Lemey P. , et al.Synchrony of Bird Migration With Global Dispersal of Avian Influenza Reveals Exposed Bird Orders, Nature Communications. (2024) 15, no. 1, 10.1038/s41467-024-45462-1, 1126. PMC10847442 38321046

[21] Mundkur T. and Langendoen T. , The First Conservation Status Review for East Asian-Australasian Flyway Now Published for Stronger Waterbirds Conservation, 2022, East Asian–Australasian Flyway Partnership (EAAFP).

[22] Zhang P. and MacIntyre C. R. , An Overview of HPAI H5N1 Clade 2.3.4.4b and Its Emerging Threat in Mainland Australia: Identified Knowledge Gaps, One Health. (2026) 22, 10.1016/j.onehlt.2025.101292, 101292. 41531463 PMC12794065

[23] Lee J. K. , Kim M. B. , and Kim S. H. , et al.Surveillance of Avian Influenza Viruses in Migratory Wild Birds in South Korea, 2019–2025, Journal of Veterinary Science. (2026) 27, no. 2, 10.4142/jvs.25249, e8. 41947677 PMC13062435

[24] van den Brand J. M. A. , Krone O. , and Wolf P. U. , et al.Host-Specific Exposure and Fatal Neurologic Disease in Wild Raptors From Highly Pathogenic Avian Influenza Virus H5N1 During the 2006 Outbreak in Germany, Veterinary Research. (2015) 46, no. 1, 10.1186/s13567-015-0148-5, 24. 25879698 PMC4349770

[25] Günther A. , Krone O. , and Globig A. , et al.Avian Raptors Are Indicator Species and Victims of High Pathogenicity Avian Influenza Virus HPAIV H5N1 (Clade 2.3.4.4b) in Germany, Scientific Reports. (2024) 14, no. 1, 10.1038/s41598-024-79930-x, 28779. 39567708 PMC11579473

[26] Ringenberg J. M. , Weir K. , and Humberg L. , et al.Prevalence of Avian Influenza Virus in Atypical Wild Birds Host Groups During an Outbreak of Highly Pathogenic Strain EA/AM H5N1, Transboundary and Emerging Diseases. (2024) 2024, no. 1, 10.1155/2024/4009552, 4009552. 40303166 PMC12016917

[27] Reading R. P. , Azua J. , and Garrett T. , et al.Differential Movement of Adult and Juvenile Cinereous Vultures (*Aegypius monachus*) (Accipitriformes: Accipitridae) in Northeast Asia, Journal of Asia-Pacific Biodiversity. (2020) 13, no. 2, 156–161, 10.1016/j.japb.2020.01.004.

[28] Kang J.-H. , Hyun B.-R. , and Kim I. K. , et al.Movement and Home Range of Cinereous Vulture *Aegypius monachus* During the Wintering and Summering Periods in East Asia, Turkish Journal of Zoology. (2019) 43, no. 3, 305–313, 10.3906/zoo-1807-3.

[29] Kim H. K. , Kim H.-J. , and Noh J. Y. , et al.Serological Evidence of H5-Subtype Influenza a Virus Infection in Indigenous Avian and Mammalian Species in Korea, Archives of Virology. (2018) 163, no. 3, 649–657, 10.1007/s00705-017-3655-z. 29204739

[30] BirdLife International , Aegypius monachus, 2021, 10.2305/IUCN.UK.2021-3.RLTS.T22695231A154915043.en.

[31] Seo Y.-R. , Lee S.-H. , and Jeong S. , et al.Genetic and Pathological Analysis of Hooded Cranes (*Grus monacha*) Naturally Infected With Clade 2.3.4.4b Highly Pathogenic Avian Influenza H5N1 Virus in South Korea in the Winter of 2022, Frontiers in Veterinary Science. (2024) 11, 10.3389/fvets.2024.1499440, 1499440. 39568482 PMC11576466

[32] Hoffmann B. , Hoffmann D. , Henritzi D. , Beer M. , and Harder T. C. , Riems Influenza A Typing Array (RITA): An RT-qPCR-Based Low Density Array for Subtyping Avian and Mammalian Influenza A Viruses, Scientific Reports. (2016) 6, no. 1, 10.1038/srep27211, 27211. 27256976 PMC4891686

[33] Zhou B. , Donnelly M. E. , and Scholes D. T. , et al.Single-Reaction Genomic Amplification Accelerates Sequencing and Vaccine Production for Classical and Swine Origin Human Influenza A Viruses, Journal of Virology. (2009) 83, no. 19, 10309–10313, 10.1128/JVI.01109-09. 19605485 PMC2748056

[34] Giussani E. , Sartori A. , and Salomoni A. , et al.FluMut: A Tool for Mutation Surveillance in Highly Pathogenic H5N1 Genomes, Virus Evolution. (2025) 11, no. 1, 10.1093/ve/veaf011, veaf011. 40093844 PMC11908534

[35] Li W. and Godzik A. , Cd-Hit: A Fast Program for Clustering and Comparing Large Sets of Protein or Nucleotide Sequences, Bioinformatics. (2006) 22, no. 13, 1658–1659, 10.1093/bioinformatics/btl158. 16731699

[36] Price M. N. , Dehal P. S. , and Arkin A. P. , FastTree: Computing Large Minimum Evolution Trees With Profiles Instead of a Distance Matrix, Molecular Biology and Evolution. (2009) 26, no. 7, 1641–1650, 10.1093/molbev/msp077. 19377059 PMC2693737

[37] Suchard M. A. , Weiss R. E. , and Sinsheimer J. S. , Bayesian Selection of Continuous-Time Markov Chain Evolutionary Models, Molecular Biology and Evolution. (2001) 18, no. 6, 1001–1013, 10.1093/oxfordjournals.molbev.a003872. 11371589

[38] Minin V. N. , Bloomquist E. W. , and Suchard M. A. , Smooth Skyride Through a Rough Skyline: Bayesian Coalescent-Based Inference of Population Dynamics, Molecular Biology and Evolution. (2008) 25, no. 7, 1459–1471, 10.1093/molbev/msn090. 18408232 PMC3302198

[39] Rambaut A. , Drummond A. J. , Xie D. , Baele G. , Suchard M. A. , and Susko E. , Posterior Summarization in Bayesian Phylogenetics Using Tracer 1.7, Systematic Biology. (2018) 67, no. 5, 901–904, 10.1093/sysbio/syy032. 29718447 PMC6101584

[40] Katoh K. and Standley D. M. , MAFFT Multiple Sequence Alignment Software Version 7: Improvements in Performance and Usability, Molecular Biology and Evolution. (2013) 30, no. 4, 772–780, 10.1093/molbev/mst010. 23329690 PMC3603318

[41] Stamatakis A. , RAxML Version 8: A Tool for Phylogenetic Analysis and Post-Analysis of Large Phylogenies, Bioinformatics. (2014) 30, no. 9, 1312–1313, 10.1093/bioinformatics/btu033. 24451623 PMC3998144

[42] Nguyen L.-T. , Schmidt H. A. , von Haeseler A. , and Minh B. Q. , IQ-TREE: A Fast and Effective Stochastic Algorithm for Estimating Maximum-Likelihood Phylogenies, Molecular Biology and Evolution. (2015) 32, no. 1, 268–274, 10.1093/molbev/msu300. 25371430 PMC4271533

[43] Yu G. , Smith D. K. , Zhu H. , Guan Y. , Lam T. T.-Y. , and McInerny G. , GGTREE: An R Package for Visualization and Annotation of Phylogenetic Trees With Their Covariates and Other Associated Data, Methods in Ecology and Evolution. (2017) 8, no. 1, 28–36, 10.1111/2041-210X.12628.

[44] R Core Team , R: A Language and Environment for Statistical Computing, 2021, R Foundation for Statistical Computing.

[45] Seok S. H. , Jeong D. H. , Park S. J. , Lee S. Y. , Lee H. C. , and Yeon S. C. , Hematologic and Plasma Biochemical Values of Cinereous Vulture (*Aegypius monachus*), Journal of Zoo and Wildlife Medicine. (2017) 48, no. 2, 514–517, 10.1638/2016-0098R1.1. 28749287

[46] Hu M. , Yuan S. , and Zhang K. , et al.PB2 Substitutions V598T/I Increase the Virulence of H7N9 Influenza A Virus in Mammals, Virology. (2017) 501, 92–101, 10.1016/j.virol.2016.11.008. 27889648

[47] Suttie A. , Deng Y.-M. , Greenhill A. R. , Dussart P. , Horwood P. F. , and Karlsson E. A. , Inventory of Molecular Markers Affecting Biological Characteristics of Avian Influenza A Viruses, Virus Genes. (2019) 55, no. 6, 739–768, 10.1007/s11262-019-01700-z. 31428925 PMC6831541

[48] Wang C. , Lee H. H. Y. , Yang Z. F. , Mok C. K. P. , Zhang Z. , and Pöhlmann S. , PB2-Q591K Mutation Determines the Pathogenicity of Avian H9N2 Influenza Viruses for Mammalian Species, PLoS ONE. (2016) 11, no. 9, 10.1371/journal.pone.0162163, e0162163. 27684944 PMC5042486

[49] Subbarao E. K. , London W. , and Murphy B. R. , A Single Amino-Acid in the Pb2-Gene of Influenza-A Virus is a Determinant of Host Range, Journal of Virology. (1993) 67, no. 4, 1761–1764, 10.1128/jvi.67.4.1761-1764.1993. 8445709 PMC240216

[50] Gabriel G. , Herwig A. , Klenk H.-D. , and Kawaoka Y. , Interaction of Polymerase Subunit PB2 and NP With Importin α1 is a Determinant of Host Range of Influenza A Virus, PLoS Pathogens. (2008) 4, no. 2, 10.1371/journal.ppat.0040011, e11. 18248089 PMC2222953

[51] Li J. W. , Ishaq M. , and Prudence M. , et al.Single Mutation at the Amino Acid position 627 of PB2 That Leads to Increased Virulence of an H5N1 Avian Influenza Virus During Adaptation in Mice Can Be Compensated by Multiple Mutations at Other Sites of PB2, Virus Research. (2009) 144, no. 1-2, 123–129, 10.1016/j.virusres.2009.04.008. 19393699

[52] Elgendy E. M. , Arai Y. , and Kawashita N. , et al.Identification of Polymerase Gene Mutations That Affect Viral Replication in H5N1 Influenza Viruses Isolated From Pigeons, Journal of General Virology. (2017) 98, no. 1, 6–17, 10.1099/jgv.0.000674. 27926816

[53] Fan S. , Deng G. , and Song J. , et al.Two Amino Acid Residues in the Matrix Protein M1 Contribute to the Virulence Difference of H5N1 Avian Influenza Viruses in Mice, Virology. (2009) 384, no. 1, 28–32, 10.1016/j.virol.2008.11.044. 19117585

[54] Song J. , Feng H. , and Xu J. , et al.The PA Protein Directly Contributes to the Virulence of H5N1 Avian Influenza Viruses in Domestic Ducks, Journal of Virology. (2011) 85, no. 5, 2180–2188, 10.1128/JVI.01975-10. 21177821 PMC3067757

[55] Song J. , Xu J. , Shi J. , Li Y. , and Chen H. , Synergistic Effect of S224P and N383D Substitutions in the PA of H5N1 Avian Influenza Virus Contributes to Mammalian Adaptation, Scientific Reports. (2015) 5, no. 1, 10.1038/srep10510. PMC4441148 26000865

[56] Yamayoshi S. , Yamada S. , and Fukuyama S. , et al.Virulence-Affecting Amino Acid Changes in the PA Protein of H7N9 Influenza A Viruses, Journal of Virology. (2014) 88, no. 6, 3127–3134, 10.1128/JVI.03155-13. 24371069 PMC3957961

[57] Gao Y. , Zhang Y. , and Shinya K. , et al.Identification of Amino Acids in HA and PB2 Critical for the Transmission of H5N1 Avian Influenza Viruses in a Mammalian Host, PLoS Pathogens. (2009) 5, no. 12, 10.1371/journal.ppat.1000709, e1000709. 20041223 PMC2791199

[58] Wang W. , Lu B. , and Zhou H. , et al.Glycosylation at 158N of the Hemagglutinin Protein and Receptor Binding Specificity Synergistically Affect the Antigenicity and Immunogenicity of a Live Attenuated H5N1 A/Vietnam/1203/2004 Vaccine Virus in Ferrets, Journal of Virology. (2010) 84, no. 13, 6570–6577, 10.1128/JVI.00221-10. 20427525 PMC2903256

[59] Stevens J. , Blixt O. , Tumpey T. M. , Taubenberger J. K. , Paulson J. C. , and Wilson I. A. , Structure and Receptor Specificity of the Hemagglutinin From an H5N1 Influenza Virus, Science. (2006) 312, no. 5772, 404–410, 10.1126/science.1124513. 16543414

[60] Wessels U. , Abdelwhab E. M. , and Veits J. , et al.A Dual Motif in the Hemagglutinin of H5N1 Goose/Guangdong-Like Highly Pathogenic Avian Influenza Virus Strains is Conserved From Their Early Evolution and Increases Both Membrane Fusion pH and Virulence, Journal of Virology. (2018) 92, no. 17, 10.1128/JVI.00778-18. PMC6096801 29899102

[61] Guo H. , de Vries E. , and McBride R. , et al.Highly Pathogenic Influenza A(H5Nx) Viruses With Altered H5 Receptor-Binding Specificity, Emerging Infectious Diseases. (2017) 23, no. 2, 220–231, 10.3201/eid2302.161072. 27869615 PMC5324792

[62] Nao N. , Kajihara M. , and Manzoor R. , et al.A Single Amino Acid in the M1 Protein Responsible for the Different Pathogenic Potentials of H5N1 Highly Pathogenic Avian Influenza Virus Strains, PLoS ONE. (2015) 10, no. 9, 10.1371/journal.pone.0137989, e0137989. 26368015 PMC4569272

[63] Jiao P. , Tian G. , and Li Y. , et al.A Single-Amino-Acid Substitution in the NS1 Protein Changes the Pathogenicity of H5N1 Avian Influenza Viruses in Mice, Journal of Virology. (2008) 82, no. 3, 1146–1154, 10.1128/JVI.01698-07. 18032512 PMC2224464

[64] Ayllon J. , Domingues P. , and Rajsbaum R. , et al.A Single Amino Acid Substitution in the Novel H7N9 Influenza a Virus NS1 Protein Increases CPSF30 Binding and Virulence, Journal of Virology. (2014) 88, no. 20, 12146–12151, 10.1128/JVI.01567-14. 25078692 PMC4178744

[65] Li J. , Zhang K. , and Chen Q. , et al.Three Amino Acid Substitutions in the NS1 Protein Change the Virus Replication of H5N1 Influenza Virus in Human Cells, Virology. (2018) 519, 64–73, 10.1016/j.virol.2018.04.004. 29677653

[66] Long J.-X. , Peng D.-X. , Liu Y.-L. , Wu Y.-T. , and Liu X.-F. , Virulence of H5N1 Avian Influenza Virus Enhanced by a 15-Nucleotide Deletion in the Viral Nonstructural Gene, Virus Genes. (2008) 36, no. 3, 471–478, 10.1007/s11262-007-0187-8. 18317917

[67] Jackson D. , Hossain M. J. , Hickman D. , Perez D. R. , and Lamb R. A. , A New Influenza Virus Virulence Determinant: The NS1 Protein Four C-Terminal Residues Modulate Pathogenicity, Proceedings of the National Academy of Sciences. (2008) 105, no. 11, 4381–4386, 10.1073/pnas.0800482105. PMC2393797 18334632

[68] Spesock A. , Malur M. , and Hossain M. J. , et al.The Virulence of 1997 H5N1 Influenza Viruses in the Mouse Model Is Increased by Correcting a Defect in Their NS1 Proteins, Journal of Virology. (2011) 85, no. 14, 7048–7058, 10.1128/JVI.00417-11. 21593152 PMC3126612

[69] Wünschmann A. , Franzen-Klein D. , Torchetti M. , Confeld M. , Carstensen M. , and Hall V. , Lesions and Viral Antigen Distribution in Bald Eagles, Red-Tailed Hawks, and Great Horned Owls Naturally Infected With H5N1 Clade 2.3.4.4b Highly Pathogenic Avian Influenza Virus, Veterinary Pathology. (2024) 61, no. 3, 410–420, 10.1177/03009858231222227. 38197395

[70] Martí-Garcia B. , Lean F. Z. X. , Núñez A. , and Majó N. , Natural Infections of Highly Pathogenic Avian Influenza Virus H5N1 in Wild Birds Between 2020 and 2023 in the UK: A Retrospective Study With Focus on Microscopic Lesions, Viral Distribution and Neurotropism, Veterinary Research. (2025) 56, no. 1, 10.1186/s13567-025-01656-z. PMC12625443 41254765

[71] Fujimoto Y. , Ogasawara K. , and Isoda N. , et al.Experimental and Natural Infections of White-Tailed Sea Eagles (*Haliaeetus albicilla*) With High Pathogenicity Avian Influenza Virus of H5 Subtype, Frontiers in Microbiology. (2022) 13, 10.3389/fmicb.2022.1007350. PMC9574225 36262320

[72] Si Y.-J. , Kim D.-J. , and Lee S.-H. , et al.New Incursions of H5N1 Clade 2.3.4.4b Highly Pathogenic Avian Influenza Viruses in Wild Birds, South Korea, October 2024, Frontiers in Veterinary Science. (2025) 11, 10.3389/fvets.2024.1526118, 1526118. 39867599 PMC11758627

[73] Takadate Y. , Mine J. , and Tsunekuni R. , et al.Genetic Diversity of H5N1 and H5N2 High Pathogenicity Avian Influenza Viruses Isolated From Poultry in Japan During the Winter of 2022–2023, Virus Research. (2024) 347, 10.1016/j.virusres.2024.199425, 199425. 38906223 PMC11250885

[74] Lee K. , Yeom M. , and Vu T. T. H. , et al.Characterization of Highly Pathogenic Avian Influenza A (H5N1) Viruses Isolated From Cats in South Korea, 2023, Emerging Microbes & Infections. (2024) 13, no. 1, 10.1080/22221751.2023.2290835, 2290835. 38044871 PMC10810616

[75] Hew Y. L. , Guinat C. , and Couty M. , et al.Introduction and Inter-Species Transmission Dynamics of High Pathogenicity Avian Influenza H5N1 Viruses in Japan 2021–25, Virus Evolution. (2026) 12, no. 1, 10.1093/ve/veag005. PMC12908673 41705171

[76] Si Y.-J. , Lee S.-H. , and Kim D.-J. , et al.First Detection of Clade 2.3.4.4b H5N1 Highly Pathogenic Avian Influenza Virus in a Wild Leopard Cat (*Prionailurus bengalensis*) in South Korea, Frontiers in Veterinary Science. (2025) 12, 10.3389/fvets.2025.1638067, 1638067. 40800228 PMC12341388

